# Editorial: Insights in food microbiology: 2021

**DOI:** 10.3389/fmicb.2022.1068066

**Published:** 2022-11-08

**Authors:** Laurent Dufossé, Dario De Medici

**Affiliations:** ^1^ESIROI agroalimentaire, Université de la Réunion, Saint-Denis, France; ^2^Microbiological Foodborne Hazard Unit, National Institute of Health (ISS), Rome, Italy

**Keywords:** food, microbiology, safety, human health, new trends

This Research Topic is part of the Insights in Frontiers in Microbiology series launched in 2021. As we are entering the third decade of the twenty-first century, and, especially in the last years, the achievements made by scientists in the field of Microbiology have been exceptional, leading to major advancements. Frontiers has organized a series of Research Topics to highlight the latest advancements in science in order to be at the forefront of science in different fields of research. This specific Editorial initiative was focused on new insights, novel developments, current challenges, latest discoveries, recent advances, and future perspectives in the field.

The Research Topic solicited brief, forward-looking contributions from the Editorial board members that describe the state of the art, outlining recent developments and major achieved accomplishments, future challenges and how to address those challenges to move the field forward. Reviews, Mini-Reviews, Perspectives, and Opinions summarizing the current state and future directions of the field were particularly welcome in this Research Topic. This Research Topic aimed to inspire, inform, and provide direction and guidance to researchers in the field.

We are pleased to note that our Research Topic has attracted contributions from many highly regarded researchers deeply involved for many years in Food Microbiology around the world, including from Austria, Brazil, Canada, China, Egypt, Finland, India, Italy, Poland, Portugal, and USA. We received 15 submissions, 13 of which were accepted (10 original research articles, two reviews, one mini-review) for publication after rigorous peer-reviews, with a total of 90 authors.

As usual with food microbiology, articles deal with good and beneficial aspects of microorganisms in food, when others list and investigate detrimental effects of unwanted microorganisms in food. Among the list of microorganisms under scrutiny, we can list the followings (non-exhaustive list): *Salmonella, Shigella*, Shiga toxins producing *Escherichia coli (STEC), Yersinia enterocolitica, Listeria monocytogenes*, and *Staphylococcus aureus*.

The fermentation of foodstuffs is one of the foundations for the past, current and future developments of humanity on the planet Earth. The literature review by Skowron et al. first lists all the advantages of fermented products (extension of the shelf life of food, inhibition of the growth of pathogenic microorganisms, improvement of the organoleptic properties, enhancement of product digestibility…). Fermentation of food can also be a source of risk if carried out under uncontrolled conditions (presence of unwanted, pathogenic microorganisms, enterotoxigenic or enterohemorrhagic bacteria). Even after millennia of practice, much remains to be done to ensure that all countries in the world provide healthy fermented products to their populations.

As a continuation of the previous work, Ghatani et al. investigated soft chhurpi, a traditionally Hymalayan Yak fermented milk, prepared by the indigenous community of Sikkim Himalayas. The two main strains found in this fermented product, *Enterococcus durans* and *Enterococcus lactis* present probiotic aspects, such as hypocholesterolemic activity and tolerance to bile salts and acid pH. It is essential for humanity to continue to isolate microbial strains with pro-health effect, from sea level to high mountain altitudes, from oceans to primary forests.

Even if current food research trends are pushing toward insect proteins, cellular meat or meat made up of 100% plant proteins, animal proteins are still widely consumed, and everyone is now aware of the intricacy between animal health and human health (see also the One-Health concept). Monte et al. conducted a large study on the biodiversity of multidrug-resistant *Salmonella* Heidelberg strains isolated from the poultry production chain across Brazil, one the largest poultry producer in the world. Authors emphasized the need for continuous mitigation programs to monitor the dissemination of this high-priority pathogen, combining molecular techniques such as WGS and CRISPR-based genotyping.

Pigs are another large source of animal proteins in the world. Large-scale animal production, often synonymous with many animals on a small surface area, induces pathologies treated with antibiotics. The use, abuse, and inappropriate use of antibiotics in livestock farms can lead to the antimicrobial resistance (AMR) in different microorganisms. Koskinen et al. defined the AMR of 1,016 pathogenic porcine *Yersinia enterocolitica* 4/O:3 strains originating from the United Kingdom, Belgium, Germany, Italy, Russia, Spain, Finland, Latvia, and Estonia. Among the conclusions of these authors, the health situation varies from country to country. More antibiotics for animal health are imported and used by the country and more the AMR increases. Parenteral medications should be preferred to orally administered mass medications, and the prudent use of antimicrobials is essential to control AMR at the farm level.

Microorganisms that are pathogenic to humans can be spread in animal husbandry but can also be present in the food processing chain. Sometimes food factories are huge and a contamination on a slicing line can cause thousands of food poisoning cases. Spampinato et al. surveyed the microbial load and the chemical–physical features of cooked hams, in modified atmosphere packaging (MAP), from five Italian producers which were monitored for a period of 12 days after the opening of the packages (i.e., the secondary shelf life). A whole set of techniques (sensorial properties, volatile metabolites, microbiota monitored by 16S ribosomal RNA gene profiling, and culture-dependent techniques…) allowed the authors to make some recommendations to the food industry. The current period tends to prohibit the use of nitrites in charcuterie and this type of study is therefore of major importance.

Raw milk, consumed as is, or used to prepare cheeses or other dairy products without any heat treatment, is also a microbiologically sensitive food. Oliveira et al. explored the prevalence and genetic diversity of *Staphylococcus aureus* and staphylococcal enterotoxins (SEs) in raw milk from the main dairy region of mainland Portugal. Recommendation is done to develop a broader SEs screening in food safety control as the majority of enterotoxigenic isolates were found to contain genes encoding SEs (SEG, SEH, and SEI) not routinely screened.

Another well-known bacterium, *Listeria monocytogenes* is difficult to control along the whole food production chain. Only long-term studies are indicated to understand how some strains are able to survive and spread as others “disappear.” Gattuso et al. reported the complete sequences of 132 clinical strains, sequences that were used to define the evolutionary relatedness among subtypes of *L. monocytogenes* isolated in Italy from 2010 to 2016. Authors stressed that phylogenic studies, based on *Listeria monocytogenes* whole-genome sequence data, using the core genome multilocus sequence type, are able to identify the emergence of highly persistent pathogenic variants, contributing to the improvement of the human hazard characterization of *L. monocytogenes*.

Listing microbial threats to animals and humans is an important task. Finding ways to better control or defeat them is also of crucial importance.

Bacteriophages are beginning to be used in some parts of the world to fight bacterial infections in humans, especially when no active antibiotics are available. Rogovski et al. propose to use the bacteriophages as bacterial control tools and environmental safety indicators in a food chain context. The ecology of the food chain is a process that is very sensitive to the environment, the microorganisms present and their succession over time. In the past, mistakes have been made, for example in trying to have zero microbial life in processes, thus creating a virgin field for contaminants. Many additional studies will be required in the coming years in order to fully appreciate the benefits of using bacteriophages in the food chain.

The elimination of (pathogenic) microorganisms by disinfectants in food processing plants is desirable and mandatory. However, it is confronted with the appearance of increasing resistance. Gundolf et al. used ionic liquids (ILs), considered as a new class of promising antimicrobials, which have been reported to be effective against resistant strains as they interact with bacterial cells in multiple ways. Structure–activity relationships, side-chain effects, cationic head groups, impact on multidrug efflux pumps were among the characteristics under study.

Food microbiology is also a question of (i) detection of microbial pathogens in a large variety of foods and (ii) development of accurate predictive models for growth, survival, or death of these microbial pathogens, in a large variety of foods, too. The detection of foodborne pathogens is increasingly based on molecular techniques but older techniques, well-established techniques are still relevant and interesting. As an example, McMahon et al. demonstrated that microbial antagonism may occur in food-enrichment culture, resulting in inhibition of Shiga toxin-producing *Escherichia coli* (STEC) and *Shigella* species. The impressive production of antimicrobial compounds in cell-free extracts from 200 bacterial strains and 332 food-enrichment broths was assessed in this paper. Considering all the results presented, the recovery of some foodborne pathogens, such as *Shigella sonnei* is an important challenge for food microbiologists and technicians.

Predicting the number of food microorganisms through modeling is a fast-growing research area. Models presented are numerous and adequation with some foods were demonstrated. Li et al. developed a Dimensional Analysis Model (DAM) they applied for *Pseudomonas* in Niuganba, a traditional Chinese fermented dry-cured beef. The study showed that the DAM model was a simple, unified and effective model to predict the number of microorganisms and storage time.

At the end of the presentation of the Research Topic content, we are back to fermentation of food, to the use of fermentation in order to produce useful food and feed ingredients. For thousands of years, humans have fermented various agricultural products to produce alcohol. This is spread all around the planet.

In their experiments, about a strong aromatic liquor, also known as strong aromatic Baijiu in China, Tong et al. described the diversity, functionality, and influence of *Bacillaceae* in the process of this beverage. Multi-microbe mixing and cooperative fermentation process are the key points.

Also initiated in Asia, a few thousand years ago, *Monascus* pigments are spreading in all the world, year after year, and the use of such pigments in food will soon increase with the development of mycotoxin-free, i.e., citrinin-free filamentous fungi strains. Abdel-Raheam et al., instead of using steamed-rice, as usually done, chose to produce pigments on a food waste, generated during potato chips manufacturing. The fungal pigments biosynthesized were then incorporated as coloring agents for ice lollies, with high acceptability from consumers.

This Editorial summarizes the articles published in this Research Topic. We hope that this Research Topic of articles will contribute to the advancement of research in Food Microbiology. Feeding the world's inhabitants without depleting the resources of the planet is a major goal for humanity.

Finally, we want to thank all the authors who contributed their original work to our Research Topic and the reviewers for their valuable comments. We would like to express our sincere gratitude to the Editorial office of Frontiers in Microbiology for their excellent support and for providing us with this opportunity to successfully conduct this Research Topic.

## Author contributions

LD drafted the manuscript. LD and DD revised the draft. All authors made a direct and intellectual contribution to the work and approved the final version for publication.

## Conflict of interest

The authors declare that the research was conducted in the absence of any commercial or financial relationships that could be construed as a potential conflict of interest.

## Publisher's note

All claims expressed in this article are solely those of the authors and do not necessarily represent those of their affiliated organizations, or those of the publisher, the editors and the reviewers. Any product that may be evaluated in this article, or claim that may be made by its manufacturer, is not guaranteed or endorsed by the publisher.

